# Protein expression of PKCZ (Protein Kinase C Zeta), Munc18c, and Syntaxin-4 in the insulin pathway in endometria of patients with polycystic ovary syndrome (PCOS)

**DOI:** 10.1186/1477-7827-10-17

**Published:** 2012-03-05

**Authors:** Rodrigo Rivero, Claire-Alix Garin, Paulina Ormazabal, Andrea Silva, Rodrigo Carvajal, Fernando Gabler, Carmen Romero, Margarita Vega

**Affiliations:** 1 Endocrinology and Reproductive Biology Laboratory, Clinical Hospital University of Chile (HCUCH), Santiago, Chile; 2 Department of Obstetrics and Gynaecology, School of Medicine, University of Chile, Santiago, Chile; 3 Department of Pathology, School of Medicine, University of Chile, Santiago, Chile

**Keywords:** PKC Zeta, Munc18c, Endometrium, PCOS

## Abstract

**Background:**

Polycystic Ovary Syndrome (PCOS) is an endocrine-metabolic disorder commonly associated with insulin resistance (IR). Previous studies indicate about the expression of molecules involved in the insulin pathway in endometria of women with PCOS-IR. Therefore, the aim of the present study was to evaluate the effect of insulin and testosterone in the expression of these proteins in the endometria and immortal endometrial stromal cell line (T-HESCs).

**Methods:**

We examined the protein levels of Munc18c, PKC zeta, phospho-PKC Zeta, and Syntaxin-4. Protein levels were assessed by Western Blot and/or immunohistochemistry in proliferative endometria (NPE = 6) and in PCOS endometria with insulin resistance (PCOSE-IR = 6). We also evaluated whether high concentrations of insulin (100 nM) and/or testosterone (100 nM), during a 24 h stimulatory period, affected the expression of these proteins in an immortal endometrial stromal cell line (T-HESCs). Once stimulated, proteins were extracted from cells and were assessed by Western Blot analysis. Immunocytochemistry was performed to detect AR in T-HESC cells.

**Results:**

Western Blot data showed decreased expression (*p *< 0,05) of Munc18c and phospho-PKC Zeta in PCOS-IR endometria (PCOSE-IR) with respect to the control (NPE). In the *in vitro *study, Western Blot analysis showed decreased levels of Munc18c, PKC Zeta and phospho-PKC Zeta with the different hormonal treatments when compared to the control condition (no hormonal stimulation) (*p *< 0,05). The AR was present in the endometrial stromal cell line (T-HESC).

**Conclusion:**

The conditions of hyperinsulinism and hyperandrogenism present in PCOS-IR patients modulate the expression and/or phosphorylation of the proteins involved in the insulin pathway at the endometrial level. These data extend to the T-HESCs cells results, where insulin and testosterone exert an effect on both the expression and phosphorylation of proteins present in the pathway.

## Background

Polycystic Ovary Syndrome (PCOS) is a common endocrine disease with an unknown etiology that affects between 5 to 10% of women in reproductive age. The principal clinical manifestations of PCOS are: oligo-anovulation, clinical and/or biochemical hyperandrogenism, and polycystic ovaries detected by ultrasonography. PCOS is associated with defects in insulin activity, where a high percentage of patients present symptoms of insulin resistance (IR), often associated with hyperinsulinemia [1]. Fat and muscle tissue samples from PCOS women present an altered content and/or activation of molecules related to the metabolic insulin signaling pathway [2,3]. An adequate expression of molecules involved in glucose uptake is necessary for the maintenance of cellular function, not only in normal insulin target tissues, but also in those involved in reproduction [4].

A previous study established the presence of the insulin receptor, PKB/Akt and the insulin dependent glucose transporter GLUT4 in endometrial tissue, indicating the presence of the insulin cascade [5]. Also, it has been reported that androgen excesses influence glucose uptake in endometrial epithelial cell cultures, which cause a decrease in the expression of IRS-1 mRNA, IRS-1 and GLUT4 [1]. Furthermore, reports have indicated that rat skeletal muscle myotubes exposed to insulin and testosterone increase phosphorylation of Ser-636/639 residue in IRS-1, compared to the control condition, suggesting a link between IR and hyperandrogenism, both of which are present in PCOS-IR women [6].

The molecular pathway that transmits the insulin signal is triggered by the binding of insulin with its receptor. This initiates the Tyr phosphorylation of IRS-1, which in turn activates PI3-K and induces downstream activation of PKB/Akt and atypical PKCs, such as PKC Zeta (PKCζ) [7]. PKCζ belongs to a Ser/Thr kinase family, and once activated by PDK1 (Thr-410) it participates in the upstream Ser phosphorylation of IRS-1, which lowers the insulin signal, acting as a negative regulator [8]. Downstream, PKCζ participates in actin remodelling, allowing the translocation of GLUT4 to the plasma membrane [9,10]. Even more, reports of primary cell cultures of rat skeletal muscle have shown that an insulin stimulus causes PKCζ to associate directly with the GLUT4 vesicle, where it phosphorylates VAMP-2 and together are translocated to the plasma membrane [11].

The fusion of the GLUT4 vesicle with the plasma membrane is mediated by the SNARE complex, which is formed by VAMP2, SNAP23, and Syntaxin-4. When Munc18c binds to Syntaxin-4, it acts as a negative regulator, inhibiting the formation of the complex [12]. However, when PKCζ interacts with Munc18c, the SNARE complex is allowed to form and the GLUT4 vesicle fuses with the plasma membrane [9,10]. The incorporation of GLUT4 into the cell surface is an essential component of glucose uptake.

In our study, we employed *ex vivo *endometrial samples and *in vitro *cultured endometrial stromal cells (T-HESCs) to explore the effects of high androgen and/or insulin concentration on expression levels of certain proteins involved in the insulin pathway.

## Methods

### Materials

The polyclonal antibodies anti-Munc18-c (M7695) and anti-Syntaxin-4 (HPA001330) were purchased from SIGMA-Aldrich (USA). The polyclonal antibodies for PKCζ (ab59364) and PKCζ (phosphoT410) (ab75816) were purchased from Abcam Inc. (USA). The polyclonal AR antibody was obtained from Santa Cruz Biotechnology, Inc. (USA). The monoclonal β-actin antibody (A5441) was purchased from SIGMA-Aldrich (USA). Secondary antibodies [anti-mouse and anti-rabbit immonuglobin G (IgG)] were purchased from Amersham Biosciences (Amersham International, Piscataway, NJ, USA). The endometrial cell line was derived from stromal cells (T-HESCs) obtained from an adult woman with miomas, and was purchased from American Type Culture Collection, ATCC number: CRL-4003. Protease inhibitor cocktail was obtained from Roche Mol Biochemicals (Mannheim, Germany) and bovine serum albumin (BSA) protein assay kit from Pierce (Rockford, IL, USA); labeled streptavidin biotin kit was purchased from Dako (CA, USA). DMEM Ham's F-12 medium (D2906), puromycin, antimycotic/antibiotic, and testosterone were all obtained from SIGMA-Aldrich (USA). Insulin-Transferrin-Selenium (ITS), sterile PBS, and human recombinant insulin were all purchased from GIBCO (USA). Fetal bovine serum treated with dextran carbon was acquired from HYCLONE (USA).

### Subjects

Control endometria (NPE) were obtained from 6 healthy women with proven fertility during the proliferative phase of the menstrual cycle, at the time of hysterectomy for benign pathologies of the uterus at the University of Chile Clinical Hospital, School of Medicine. The PCOS-IR endometrial samples (PCOSE) were obtained by Pipelle suction curette from the corpus of the uteri of 6 women. The endometrial morphology of PCOS women is comparable to the proliferative phase of control women. This was the first time these women attended the Infertility Clinic at the University of Chile Clinical Hospital, School of Medicine. None of the women, neither controls nor those with PCOS, had taken oral contraceptives or other medications for at least 3 months before starting the study. Glucose and insulin levels were evaluated by an oral glucose tolerance test with a 75 g load of glucose. In order to determine an IR condition, we measured plasma glucose and insulin levels at 2 h post glucose load. The diagnosis of IR was determined when 120 min insulin levels were 2 SDS of insulin concentration over the mean of the control group, as in previous studies [5]. Additionally, homeostasis model assessment (HOMA-IR) index was calculated for all patients. Noyes criteria [13] were applied by an experienced pathologist to confirm, on the basis of histological dating, the endometrial phase of control and PCOS-IR endometria. The diagnosis of PCOS was made according to the Rotterdam Consensus [14] and the Androgen Excess Society criteria [15] for the definition of PCOS. The exclusion criteria were women who presented hyperprolactinemia (prolactin (PRL) >35 ng/ml), hypothyroidism (TSH >5 mIU/ml), androgen secreting tumors (total testosterone >2 ng/ml; dehydroepiandrosterone sulfate (DHEAS) >2600 ug/dl), Cushing's syndrome (urine cortisol concentration >50 ug/dl at 24 h and fasting plasma concentration of cortisol >25 ug/dl), congenital adrenal hyperplasia (17-OH progesterone >2,5 ng/ml), diabetes or treatment with hormones and/or ovulation induction. The reference values are from the World Health Organization Program (estradiol and progesterone), glycaemia values are a world consensus, basal insulin is based in the values stated in the kit and 120 minutes insulin level post administration of glucose and the other hormones determinations normal range values are from the Laboratory of Endocrinology and Reproductive Biology, University of Chile Clinical Hospital, School of Medicine. This investigation was approved by the Ethical Committees from the University of Chile Clinical Hospital, School of Medicine and informed written consent was obtained from all subjects.

### Cell culture and treatments

The T-HESCs cells were cultured in growth media (DMEM Ham's F-12 medium, 1X Insulin-Transferrin-Selenium (ITS), 10% fetal bovine serum treated with dextran carbon, puromycin 500 ng/mL, 1X of antimycotic/antibiotic) at 37°C in a 5% CO_2 _atmosphere until confluence was achieved. After achieving about 80% confluence, cells were cultured in 10 cm Petri-dishes for 48 h at a ratio of 800000 cells/plate in growth media without the insulin contained in ITS, at 37°C in a 5% CO_2 _atmosphere. The cultures with no hormonal stimulation were used as the control for the study. Then, cells were washed twice with sterile PBS. The cultures were further subjected for 24 h to three different treatments: 1) the addition of 100 nM testosterone; 2) 100 nM of human recombinant insulin; and 3) the addition of 100 nM testosterone and 100 nM human recombinant insulin. Once stimulated for 24 h, the cell cultures were washed with cold PBS; then, lysis buffer (25 mM Tris-HCl pH 7.4, 0.5 mM EGTA 25 mM NaCl, 1% Nonidet P-40, 1 mM Na_3_VO_4_, 10 mM NaF, 1X protease inhibitors (Roche, IN)) was added. Next, the cells were scraped and sonicated at 4°C and centrifuged at 1500 g for 6 min at 4°C. The resulting supernatant was used to determine the protein concentration with the BCA Protein Assay kit.

### Immunocytochemical detection

For immunocytochemical detection, 200000 T-HESC cells were cultured on slides for 24 h, with or without testosterone and then were permeabilized with 0.3% Triton for 10 min at room temperature. Slides were then incubated in 3% hydrogen peroxide for 15 min to prevent endogenous peroxidase activity. Next, non-specific binding was inhibited by incubating samples in non-fat dehydrated milk for 10 min. The primary antibody for AR (polyclonal, 1:150) was applied to the samples and incubated overnight at 4°C. Negative controls were analyzed on adjacent sections incubated without the antibody. The secondary antibody was an anti-rabbit IgG-HRP diluted at 1:300. Samples were incubated for 30 min at 37°C. The chromogen was developed by the streptavidin-peroxidase system and 3, 3' diaminobenzidine was used as the substrate, counterstaining was performed with hematoxylin. The slides were evaluated on an Olympus optical microscope (Olympus BX51TF Tokyo, Japan).

### Tissue preparation

Endometrial tissue samples from the two studied groups (NPE and PCOSE-IR) were divided into two fragments. A fragment of each sample was fixed in 4% buffered formaldehyde for 24 h, embedded in paraffin and cut in 5 to 6 μm thick sections before histological and immunohistochemical studies. The second fragment of each sample was first frozen in liquid nitrogen and stored at -80°C, and then homogenized in lysis buffer (25 mM Tris-HCl pH 7.4, 0.5 mM EGTA 25 mM NaCl, 1% Nonidet P-40, 1 mM Na_3_VO_4_, 10 mM NaF), and 1X protease inhibitor (Roche, IN) was then added. Afterwards, samples were centrifuged at 10000 g for 20 min at 4°C. The resulting supernatant was used to determine the protein concentration with the BCA Protein Assay kit.

### Immunohistochemical detection

Paraffin sections of human endometrial tissue were deparaffinized in xylene and gradually hydrated through graded alcohols. The sections were incubated in 10 mM sodium citrate buffer (pH 6.0) at 95°C for 30 min, incubating the samples in 3% hydrogen peroxide for 15 min prevented endogenous peroxidase activity. Nonspecific antibody binding was inhibited by incubating samples with the blocking solution (HistostainRSP, ZYMED) for 10 min. Primary antibodies PKCζ (polyclonal; 1:100), phosphorylated PKCζ (polyclonal; 1:100), Munc18c (polyclonal; 1:1000) and Syntaxin-4 (polyclonal; 1:350) were applied to the samples and incubated overnight at 4°C. The negative controls were analyzed on adjacent sections incubated without the respective primary antibody. The secondary antibody used in all cases was a biotinylated anti-rabbit/anti-mouse/anti-goat immunoglobulin. The chromogen was developed by the streptavidin-peroxidase system and 3, 3' diaminobenzidine was used as the substrate; counterstaining was performed with hematoxylin. The slides were evaluated on an Olympus optical microscope (Olympus BX51TF Tokyo, Japan). Slide analysis was performed by the measurement of positive pixel intensity with the use of the semi quantitative analysis tool IOD (Integrated Optical Density), in the Image Pro Plus 6.0 program. Equally sized areas were taken at random in the stroma and epithelia in different regions of the sample. The mean of these values was obtained per sample and studied group.

### Western blot analysis

Total protein (50 μg of endometrial tissue and 25 μg of cultured cells) was denatured and fractionated using 10% 1D-SDS-PAGE and transferred to a nitrocellulose membrane. Membranes were blocked for 1 h in TTBS (20 mM of Tris, pH 7.5; 137 mM of NaCl; and 0.1% Tween-20) with 10% non-fat dehydrated milk or 10% BSA. The membranes were washed twice for 5 min with TTBS and incubated with antibodies against Munc18c (polyclonal 1:500), PKCζ (polyclonal 1:1000), phospho-PKCζ (polyclonal 1:1000), and Syntaxin-4 (polyclonal 1: 500), overnight at 4°C. The membranes were then washed twice for 5 min with TTBS and incubated for 1 h at room temperature with anti-rabbit IgG peroxidase-linked species-specific antibody (1: 10000). After the membranes were washed with TTBS three times for 5 min, the bound antibodies were detected with an enhanced chemiluminicence system. Band intensities were quantified by scanning densitometry utilizing the UN-SCAN-IT software, Automated Digitizing System, version 5.1.

### Statistical evaluation

The number of subjects in this study was calculated assuming α = 0.05 and β = 0.20 and a difference between means of 0.25 and a standard deviation of 0.2 according to previous studies [5]. The data were analysed using the Kolmogorov-Smirnov distribution test to determine if they were parametric or not. Comparison groups were analysed by Student's *t*-test for parametric distributions, and by the Mann Whitney test for non-parametric distributions. For multiple comparisons, the ANOVA statistical test was used followed by the Tukey test. For all cases, *p*-values < 0.05 were considered significant. Statistical tests were performed using Prism for Windows, version 5.0 (Graph-Pad Software, Inc).

## Results

### Clinical and endocrine characteristics

The clinical and endocrine characteristics of the two groups of women are summarized in Table 1. The higher mean age of the NPE (Normal Proliferative Endometrium) compared to PCOS women is inherent to the design of the study, where the endometrial samples from the control group are obtained at the time of hysterectomy. All PCOS women presented a BMI compatible with the condition of moderately obesity, according to the fact that 50-70% of PCOS women are obese, while the control group was preferentially women bearing overweight. In addition, all PCOS women participants of this study presented excessive ovarian androgen production, which taking into account the decreased SHBG blood levels, leads to a significantly higher free androgen index (FAI) in these women. All women diagnosed as bearing PCOS were also classified as insulin resistant by the appropriate tests, thus grouped under PCOSE-IR (Table 2).

**Table 1:** Clinical and hormonal characteristics of the subjects

	Control NPE	PCOSE-IR
*AGE*	39.7 ± 1.2	27.2 ± 0.6*

*BMI*	29.7 ± 2	32.7 ± 0.9

*Testosterone (ng/dL)*	0.4 ± 0.1	0.8 ± 0.1*

*SHBG (nmol/L)*	51.7 ± 9.5	24.2 ± 2.1*

*FAI*	3.7 ± 0.7	13.5 ± 1.6*

*Estradiol (pmol/L)*	270.7 ± 22.4	228.3 ± 3.5

*Progesterone (nmol/L)*	2.2 ± 0.3	1.9 ± 0.1

*17-OH-Progesterone (ng/mL)*	1.4 ± 0.3	19 ± 0.2

*Androstenedione (ng/mL)*	0.6 ± 0.2	2.8 ± 0.2*

The values are expressed as mean ± SEM. *p < 0.05 NPE *vs *PCOSE-IR Blood samples from women during their normal proliferative phase (NPE) and from women with Polycystic Ovarian Syndrome and Insulin Resistance (PCOSE-IR). n = 6 for each studied group. *BMI *Body Mass Index, *FAI *Free Androgen Index, *FP *Follicular Phase, *LP *Luteal Phase

**Table 2:** Metabolic characteristics of the subjects

	NPE	PCOSE-IR
*Basal Glycaemia (mg/dL)*	66.3 ± 7.4	69.4 ± 3.0

*Glycaemia 120 min (mg/dL)*	73.5 ± 9.8	105.5 ± 6.0

*Basal Insulin (μUI/mL)*	13.6 ± 2.3	19.3 ± 3.1

*Insulin 120 min (μUI/mL)*	49.6 ± 5.8	116.5 ± 14.1*

*HOMA-IR*	2.2 ± 0.91	3.3 ± 0.6

Values are expressed as mean ± SEM. NPE = Normal Proliferative Endometrium; PCOSE-IR = women with Polycystic Ovary Syndrome with Insulin Resistance. HOMA-IR: fasting glycaemia × insulinemia divided by 405. *p < 0.05 between groups

### Immunodetection of Munc18c, PKCζ, phospho-PKCζ and Syntaxin-4

**Figure 1:**
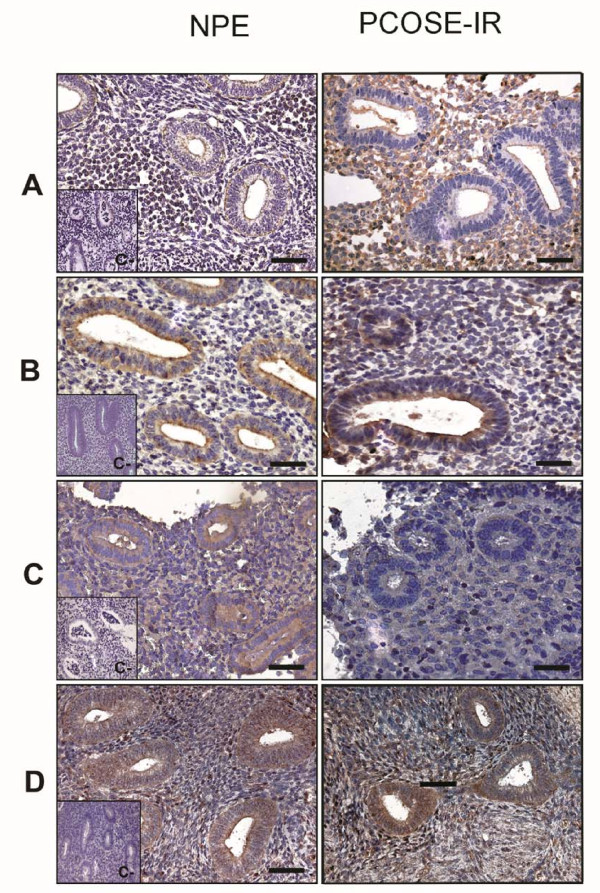
**Immunohistochemistry for proteins of the insulin signaling pathway**. Representative image from immunohistochemical detection of Munc18c (A), PKCζ (B), phospho-PKCζ (C) and Syntaxin-4 (D) in paraffin wax sections of proliferative endometria (n = 6) (left panel) and PCOSE-IR (n = 6) (right panel). The hematoxylin/eosin staining showed comparable architecture in the two analyzed groups. Positive staining was detected in epithelial and stromal cells of all studied endometria for all antigens. Magnification 400× in all panels. Inserts show negative controls for immunohistochemical assays. Scale bars represent 50 μm.

Immunolocalization of Munc18c, PKCζ, phospho-PKCζ and Syntaxin-4 in endometria obtained from the two groups of women is shown in Figure 1 and Table 3. The immunohistochemical analysis of Munc18c revealed an increase in the protein levels of PCOSE-IR when compared to NPE stromal compartment (*p *< 0.05). PKCζ protein detection showed that endometria from proliferative phase presented a higher epithelial immunostaining compared to the stromal compartment (*p *< 0.05) (Figure 1). In PCOS-IR endometria, a decrease in the detection of the protein in both compartments was observed when compared to the control endometrium in the proliferative state (*p *< 0.05). The analysis of the staining in the PCOS-IR endometria, reveals a homogeneous distribution in both compartments, epithelial and stromal. For the phosphorylated form of the protein (phospho-PKCζ), a homogeneous staining in compartment in the proliferative phase of the menstrual cycle was observed. In PCOS-IR endometria, a decrease in the expression of the protein in both compartments was noticeable, with a significant difference between the stromal compartment of the endometrium PCOS-IR compared to the same compartment in control endometrium in the proliferative state (*p *< 0,05).

**Table 3:** Inmunohistochemical detection of Munc18c, PKCζ, phospho-PKCζ and Syntaxin-4, in samples from NPE and PCOSE-IR

	EPITHELIAL COMPARTMENT (IOD)		STROMAL COMPARTMENT (IOD)	
	***NPE***	***PCOSE-IR***	***NPE***	***PCOSE-IR***

*Munc18c*	1996 ± 503.9	3347 ± 656.8	1497 ± 336.1	3380 ± 819^‡^

*PKC ζ*	8137 ± 2285	4652 ± 810.6^‡^	2646 ± 928.8*	2745 ± 665

*Phospho-PKC ζ*	4765 ± 2351	276,4 ± 34.5	3274 ± 1661	346,7 ± 35.5^‡^

*Syntaxin-4*	44882 ± 11522	29900 ± 6695^‡^	12753 ± 3576*	15818 ± 3189

The samples were semiquantified by IOD. Values are expressed as mean ± SEM. NPE = Normal Proliferative Endometrium; PCOSE-IR = women with Polycystic Ovary Syndrome with Insulin Resistance.*p < 0.05; Epithelium Compartment *vs *Stromal Compartment;‡ *p *< 0.05; NPE v/s PCOSE-IR.

Abbreviations: *AR *Androgen Receptor, *SHBG *Sex hormone-binding globulin; *FAI *Free Androgen Index; *HOMA IR *Homeostasis Model Assessment Insulin resistance

In general, Syntaxin-4 showed more immunostaining in the epithelial compartment compared to the stroma (Figure 1). Moreover, when compared to NPE a significant (*p *< 0.05) decrease in epithelial staining was observed in PCOS-IR endometria, whereas, higher IOD levels were obtained in the stromal compartment of pathological endometria. In the PCOS-IR endometria, immunostaining was similar between both compartments.

### Expression levels of proteins in endometrial tissues

**Figure 2:**
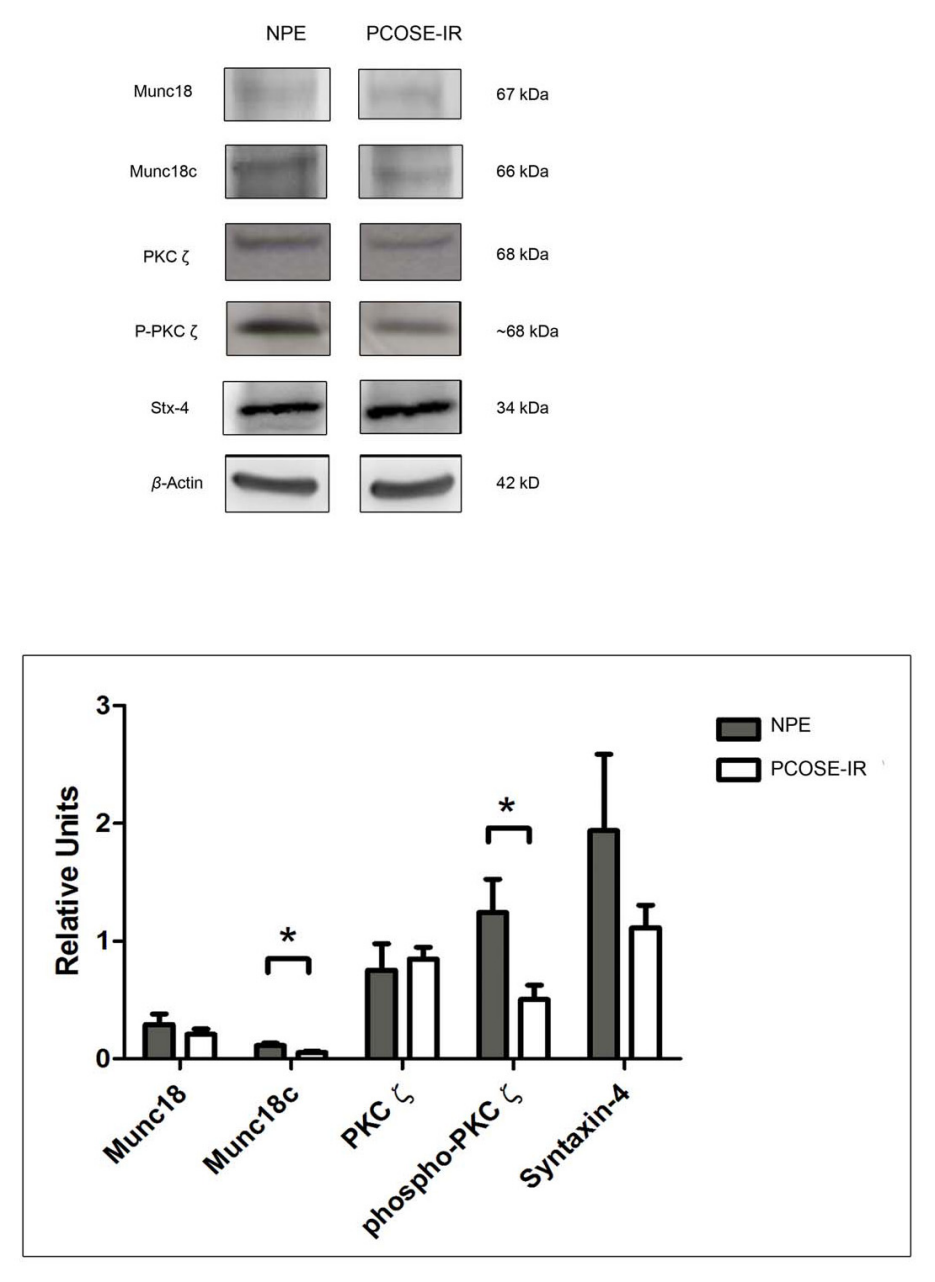
**Western blot analysis in endometria from proliferative phase (NPE) and from PCOS women with insulin resistance (PCOSE-IR)**. Equal amounts of protein were loaded in each lane. Munc18c (66 kDa), PKCζ (68 kDa), phospho-PKCζ (~68 kDa) and Syntaxin-4 (34 kDa) band intensities were quantified by scanning densitometry and normalized to intensities observed for β-actin as an internal control. A representative Western Blot for Munc18c, PKCζ, phospho-PKCζ and Syntaxin-4 is shown. The results are expressed as relative units (RU), and the values shown are mean ± SEM in Munc18c (n = 6), PKCζ (n = 6), phospho-PKCζ (n = 6) and Syntaxin-4 (n = 6). * *p *< 0.05 between Munc18c (NPE v/s PCOSE-IR) and phospho-PKCζ (NPE v/s PCOSE-IR).

Western Blot analysis was performed for Munc18c, PKCζ, phospho-PKCζ and Syntaxin-4. The analysis of these proteins was done comparing protein expression between control and PCOS-IR endometria (Figure 2). The protein expression of Munc18c decreased significantly, approximately 50%, in the PCOS-IR endometria compared to the control NPE (*p *< 0.05). On the other hand, no variation in the protein expression for PKCζ was observed. However, the phosphorylation of the protein, expressed as the ratio of phospho-PKCζ/β-actin a significant decrease (approximately 40%), with respect to the control endometria (*p *< 0.05).

### Immunocytochemical detection of AR in T-HESCs cells

**Figure 3:**
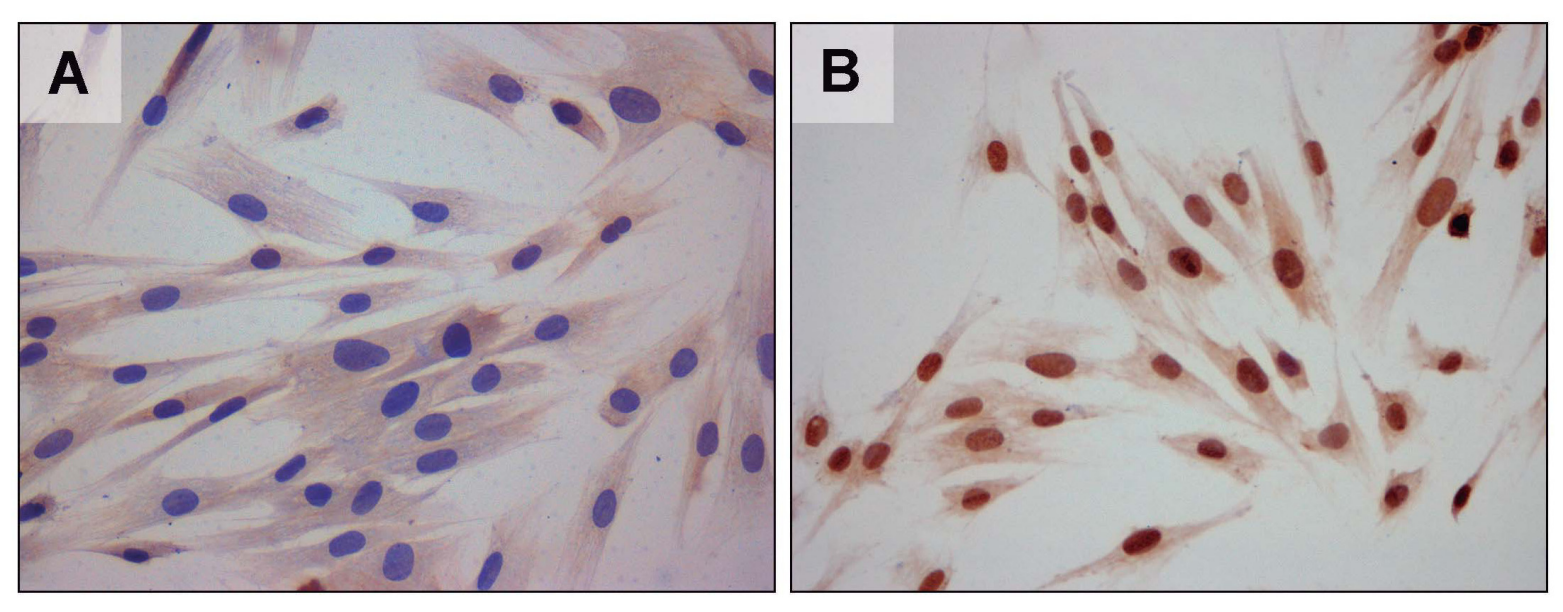
**Immunocytochemical detection of AR in T-HESCs cells**.

In unstimulated T-HESC cells, a weak immunostaining was observed for AR mostly in the cytoplasm (Figure 3A). However, when cells were stimulated with androgens, the positive staining was observed mainly at the nuclear level (Figure 3B). This is in agreement with previous observations for the AR in the endometrial tissue samples [16,17].

### Effect of insulin and/or testosterone in T-HESC cells

An endometrial stromal cell line was used to determine if hormone excess in cell culture affects protein expression in the insulin pathway. In order to simulate the PCOS-IR condition in cells, high doses of testosterone and insulin were added to the culture system.

**Figure 4:**
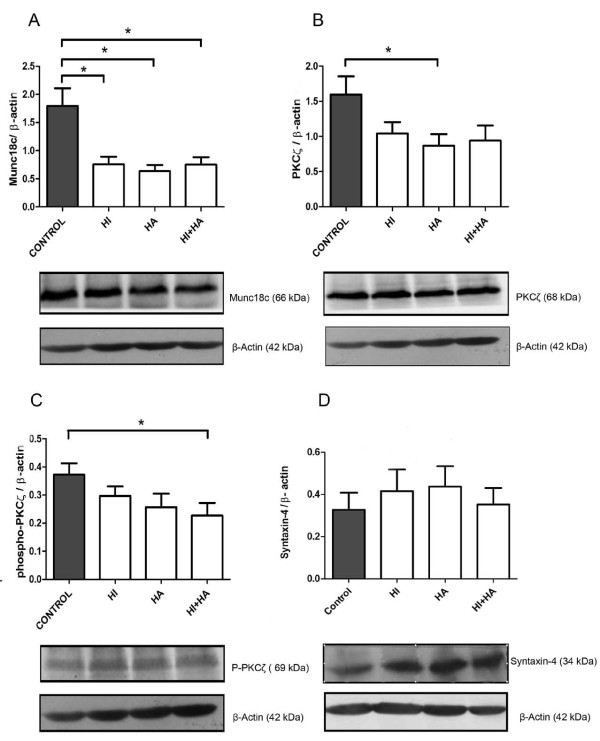
**The effects of insulin and/or testosterone on protein expression in T-HESCs cell cultures**. Protein levels were assessed in cell exposed to 100 nM insulin (HI), 100 nM testosterone (HA), 100 nM insulin plus 100 nM testosterone (HI + HA), aand control cells. Representative Western Blot is shown below the bar graphs. Equal amounts of cell lysate were loaded in each lane, all the proteins were detected according to molecular mass: Munc18c (66 kDa) (A), PKCζ (68 kDa) (B), phospho-PKCζ (~68 kDa) (C) and Syntaxin-4 (34 kDa) (D). Band intensities were quantified by scanning densitometry and normalized to intensities observed for β-Actin. Results were expressed as relative units (RU), and the values are shown as mean ± SEM, * *p *< 0.05 between control and each hormonal treatment.

Exposure of T-HESCs to 100 nM insulin and/or 100 nM testosterone significantly decreased protein expression evaluated by Western Blot of Munc18c (*p *< 0.05) (Figure 4A). PKCζ protein expression was significantly decreased when the cells were treated with 100 nM testosterone (*p *< 0.05). However, the other hormonal treatments for PCK ζ did not reach the level of statistical significance (Figure 4B). The phosphorylation of PKCζ was reduced when cells were treated with the combination of 100 nM insulin and 100 nM testosterone compared to the control condition (Figure 4C); whereas, all treatments did not affect Syntaxin-4 protein expression in cells (Figure 4D).

## Discussion

Previous studies report that the synergetic effect between the overproduction of androgens and insulin resistance present in many PCOS women contributes to the alteration of the function in several tissues, including the endometrium [2,3]. In turn, excess androgen induces changes in the expression of proteins related to tissue homeostasis, intracellular steroid bioavailability [18-20] and uterine receptivity [17] in PCOS endometria.

Also, it has been described that hyperinsulinemia in PCOS could possibly be due to defects in the expression and/or activity of proteins downstream from the insulin receptor [21,22]. Thus, understanding protein expression in insulin signaling in PCOS-IR patients could help expand knowledge about reproductive failure observed in the majority of these cases [5,23,24].

Several laboratories have established that the endometrium is an insulin responsive tissue, by indicating the presence of GLUT4 mRNA and its protein, in normal and PCOS tissue [5,25,26]. Therefore, to better understand insulin activity in this tissue, in the present investigation proteins that participate in the pathway were evaluated, specifically PI3K/PDK-1 pathway molecules that are known to have a crucial role in glucose uptake in endometrial cells tissue [5]. Our first goal was to determine the presence and cellular distribution of some key proteins involved in the translocation of GLUT4 to the plasma membrane, in both normal and PCOS endometria. The study of these molecules in the endometrium is of special importance because glucose serves as the main energy provider, and inadequate uptake into endometrial PCOS-IR cells has been often linked with failure to conceive. In this respect, one of the proteins assayed in the present investigation was PKCζ. Previous reports have shown that PKCζ participates in the remodelling of cortical actin [7], and the phosphorylation of various proteins in the insulin cascade [11]. It is important to note that phosphorylated PKCζ is lower in PCOS-IR endometria (Figure 2), suggesting a potential decreased translocation of activated PKCζ to the plasma membrane and thus probably a decreased glucose uptake in PCOS-IR endometrium [27]. It is interesting that the remodelling of cortical actin for GLUT4 vesicle translocation is also orchestrated by WAVE family proteins. In fact, a previous study has shown reduced protein levels of N-WASP and WASP in endometrial tissue of women with PCOS and hyperinsulinemia [24].

On the other hand, the diminished Munc18c expression in PCOS-IR endometria, as assessed by western blot (Figure 2), could indicate a possible failure in the interaction of this protein with phosphorylated PKCζ [9,10,28,29]. It should be mentioned that the immunodetection of Munc18c was higher in the stromal compartment on PCOS-IR endometria, we have no clear explanation for this result, but we can speculate that this discrepancy could reside in the different techniques used. Interestingly, protein levels of Syntaxin-4 were not affected by the PCOS-IR condition. Together, these data could indicate that the lowered expression of Munc18c and phosphorylated PKCζ are not directly affecting Syntaxin-4 levels, suggesting there could be other proteins responsible for the failed translocation of GLUT4 to the plasma membrane.

The results discussed up to this point, do not allow us to conclude if the condition of hyperandrogenism and/or hyperinsulinemia present in the PCOS-IR condition can affect protein levels in endometrial tissues. Therefore, in order to better understand the action of high doses of androgens and/or insulin, we employed an endometrial stromal cell line which we stimulated *in vitro *with exogenous hormones. The action of testosterone and insulin were through their specific receptors present in the T-HESC cells. The androgen receptor is located in the cytoplasm and nucleus of HESCs cells, whereas, the insulin receptor is observed on the cell surface (unpublished data). Therefore, we believe that testosterone and insulin could be acting through their specific receptors present in the cells and thus T-HESC cultures should respond to hyperandrogenic and hyperinsulinic environments.

Our results showed that high concentrations of insulin significantly affect the protein expression of Munc18c. This transient hyperinsulinic condition allows us to infer that the insulin resistant condition present in PCOS patients could be altering the expression of this protein without affecting the protein expression of Syntaxin-4, a protein regulated by Munc18c. On the other hand, the treatment with high concentrations of testosterone to the T-HESC cells decreased the levels of both phospho-PKCζ (Thr410) and Munc18c, suggesting that high levels of the hormone can participate in insulin resistance in the endometrium, which could result in disturbed glucose uptake [1].

When T-HESC cells were stimulated with both hormones, insulin and testosterone, we observed decreased protein levels of Munc18c and phospho-PKCζ. These results are in agreement with the results obtained in this investigation in PCOS-IR endometria for the same proteins. Therefore, hormone excesses characteristic of PCOS affect the expression of key proteins involved in insulin action at endometrial level. This observation suggests a lowered GLUT4 vesicle translocation to the cell periphery, eventually leading to a deficient entrance of glucose to the cell. Therefore, the defects in the insulin signaling pathway observed at the protein level, including Munc18c, PKCζ, phospho-PKCζ, and Syntaxin-4 in PCOS patients with insulin resistance, could lead to impaired glucose uptake. Accordingly, the involvement of insulin resistance and high androgen levels in the molecular defects of the insulin cascade cannot be discarded.

## Conclusions

The condition of hyperinsulinism and hyperandrogenism present in PCOS-IR patients could modulate the expression and/or the phosphorylation of proteins associated with the insulin pathway at endometrial level. This coincides with results obtained in T-HESCs cells, where insulin and testosterone exert an effect on both the expression and phosphorylation of associated proteins. In summary, hormonal imbalances in PCOS-IR patients seem to regulate protein expression, as seen in results obtained from both Western Blot and immunohistochemistry, where PCOS-IR patients seem to have lowered protein levels, all of which could potentially affect the reproduction capacity of these women.

## Competing interests

The authors declare that they have no competing interests.

## Authors' contributions

RR, CAG, PO, and MV conceived and designed the experiments. RR, CAG, PO, and AS performed the experiments; RR, CAG, PO, AS, CR and MV analyzed the data. MV and CR contributed reagents/materials/analysis tools. RR, CAG and MV wrote the manuscript. All authors read and approved the final manuscript.
